# Physiological and Proteomic Analysis of Various Priming on Rice Seed under Chilling Stress

**DOI:** 10.3390/plants13172430

**Published:** 2024-08-30

**Authors:** Hua Zhang, Guo Hui, Guoqing Gao, Izhar Ali, Maoyan Tang, Lei Chen, Xiaoyuan Zhong, Ligeng Jiang, Tianfeng Liang, Xiaoli Zhang

**Affiliations:** 1 Guangxi Key Laboratory of Rice Genetics and Breeding, Rice Research Institute, Guangxi Academy of Agricultural Sciences, Nanning 530007, China; zh347029559@163.com (H.Z.); gh8207@163.com (G.H.); gqgao@gxaas.net (G.G.); tangmaoyan@gxaas.net (M.T.); chenlei@gxaas.net (L.C.); zhongxiaoyuan2015@163.com (X.Z.); 2 Key Laboratory of Crop Cultivation and Physiology, Education Department of Guangxi Zhuang Autonomous Region, Guangxi University, Nanning 530004, China; izharali48@gmail.com (I.A.); jiang@gxu.edu.cn (L.J.); 3 College of Agronomy, Nanjing Agricultural University, Nanjing 210095, China

**Keywords:** rice, seed priming, chilling stress, proteomics response, germination

## Abstract

Rice (*Oryza sativa* L.) cultivation using direct seeding is susceptible to chilling stress, particularly during seed germination and early seedling growth in the early season of a double cropping system. Alternatively, seed priming with various plant growth-promoting hormones is an effective technique to promote rapid and uniform emergence under chilling stress. Therefore, we evaluated the impact of gibberellin A3 (GA_3_) and brassinolide (BR) priming on rice seed emergence, examining their proteomic responses under low-temperature conditions. Results indicated that GA_3_ and BR increased the seed germination rate by 22.67% and 7.33% at 72 h and 35% and 15% at 96 h compared to the control (CK), respectively. Furthermore, proteomic analysis identified 2551, 2614, and 2592 differentially expressed proteins (DEPs) in GA, BR, and CK, respectively. Among them, GA exhibited 84 upregulated and 260 downregulated DEPs, while BR showed 112 upregulated and 102 downregulated DEPs, and CK had 123 upregulated and 81 downregulated DEPs. Notably, under chilling stress, both GA_3_ and BR are involved in peroxide metabolism, phenylpropanoid biosynthesis, and inositol phosphate metabolism, enhancing antioxidant capacity and providing energy substances for germination. In addition, GA_3_ triggers the specific regulation of stress responsive protein activation, GTP activation, and ascorbic acid biosynthesis and promotes the stability and integrity of cell membranes, as well as the synthesis of cell walls, providing physical defense for seeds to resist low temperatures. At the same time, BR triggers specific involvement in ribosome synthesis and amino acid synthesis, promoting biosynthetic ability and metabolic regulation to maintain plant life activities under low-temperature stress. Furthermore, the various genes’ expression (*OsJ_16716*, *OsPAL1*, *RINO1*) confirmed GA_3_ and BR involved in peroxide metabolism, phenylpropanoid biosynthesis, and inositol phosphate metabolism, enhancing antioxidant capacity and providing energy substances for germination. This study provides valuable insights into how rice seed embryo responds to and tolerates chilling stress with GA_3_ seed priming.

## 1. Introduction

Rice (*Oryza sativa* L.) is a warm and short day crop that is highly sensitive to low temperature stress, especially during the germination stage [1]. In Guangxi province, China, rice is cultivated twice a year, with an early season and a late season. During the early season, temperatures often fall below 12 °C and continue for more than a week, significantly impacting seed germination [1], while the minimum temperature required for rice seeds to germinate is 15 °C [2]. When the average temperature drops below 15 °C on a day, the germination of rice seeds with direct seeded planting will be significantly hindered [2], leading to rice rot and delayed maturity, seriously threatening the safe production of late rice [3]. Alternatively, in many field crops, seed priming is an effective, useful, and straightforward method to promote quick and consistent emergence, high seedling vigor, and yield in adverse environmental circumstances [1,2,3]. Seed initiation is a pre-treatment method for sowing, first proposed by Heydecker et al. [4]. By using specific triggering substrates at low temperatures, the seeds can slowly absorb water and reach a germination state, without causing the embryonic roots to break through the seed coat [5,6]. This activates the repair and stress resistance metabolism regulation of the seeds in advance, significantly promoting the germination process of the seeds and alleviating the adverse effects of low-temperature stress on rice seed germination [1,7]. At the same time, seed initiation technology has significant advantages such as easy operation, low cost, high application value, and easy widespread promotion. Therefore, it has shown extremely broad application prospects in the agricultural field. Notably, our previous study underscores that seed priming with plant growth-promoting hormones emerges as the most sustainable and adaptable approach to enhance rice seed germination under cold stress [1].

Among plant growth-promoting hormones, gibberellin A3 (GA_3_) enhances rice seedling emergence and establishment by promoting mesocotyl elongation, potentially expanding the range of varieties suitable for deep direct-seeding [8]. Additionally, GA_3_ seed priming reduced ABA content and enhanced GA content in rice seedlings under low temperature, leading to decreased excessive energy generation [1,9]. Similarly, brassinolide (BR) seed priming significantly improves the seed germination rate under low-temperature stress in lucerne (*Medicago sativa* L.) [10]), *Brassica juncea* [11], *Leymus chinensis* [12], and rice [1]. Both GA_3_ and BR improved rice seedling growth, root morphology, seedling dry matter production, and physiological and biochemical indicators [1]. The difference between seed pre-treatment and seed initiation methods is due to their specific use and target. Seed pre-treatment includes various techniques, such as scarification, soaking, or chemical treatments, to improve seed germination and growth in general [8,9,10], while seed initiation technology is very useful for promoting germination in unfavorable environments like cold temperatures because it uses regulated circumstances to pre-germinate seeds until just before radicle emergence, activating stress tolerance and repair mechanisms in the process [4,5,6,7]. Previous studies primarily examined the morphological, physiological, and growth indicators of rice seedlings under cold stress with these priming agents, necessitating more detailed investigations into the cold tolerance phenomenon. The molecular mechanism for the proteomic response of rice development embryos to cold stress during seed germination remains unclear.

Proteomic analysis is vital for understanding the precise roles of proteins in seed germination and plant development [13,14]. Numerous studies, focusing on Arabidopsis, tomato, and barley seeds, have contributed to our knowledge in this area. In Arabidopsis, around 1300 total seed proteins were detected on two-dimensional (2-D) gels, with 74 proteins changing during germination, particularly at the radicle protrusion step [15]. Germinating tomato seeds revealed 47 major germination-related proteins in the embryo and endosperm [16]. For barley, 44, 42, and 19 spots were identified in the embryo, aleurone layer, and endosperm tissues, respectively [17]. A study on the proteomic analysis of germinating rice seeds using 2-DGE and mass spectrometry identified differentially expressed proteins involved in various functions, including metabolism, stress response, and detoxification, highlighting their importance during germination [16]. Furthermore, the proteomic analysis of rice seedlings shows that under stress, energy metabolism-related proteins, like Ribulose-1,5-bisphosphate carboxylase/oxygenase large subunit (*RuBisCO LSU*), increase, while defense-related proteins decrease [18]. Furthermore, Yoon reported that under stress, rice seeds upregulate proteins like Oryza sativa cytidine triphosphate synthetase1 (*OsCTPS1*) and minichromosome maintenance proteins, which are crucial for DNA replication and seed viability [19]. Hybrid rice cultivars were investigated for their development or stress-related protein profiles in rice embryo tissues using 2-D gel electrophoresis (2-DGE) [16,20]. Additionally, a proteomic analysis of rice germination under gibberellin A3 (GA_3_) and abscisic acid (ABA) modulation focused on embryonic tissue, revealing that proteins in the embryo, rather than the endosperm, are sensitive to applied phytohormones [21,22]. However, a complementary study on protein regulation in embryo tissues during germination is currently lacking.

Proteomic analysis is crucial for comprehending protein functions during seed germination and plant development stages. However, there is a lack of studies addressing protein regulation in embryo tissues, particularly during germination under cold stress with BR and GA_3_ seed priming. Therefore, this study aimed to (1) assess the influence of GA_3_ and BR seed priming on seed germination rates and (2) examine the proteomic response of rice embryos during development under cold stress.

## 2. Material and Methods

### 2.1. Experimental Design and Treatment Details

In 2022, a complete randomized design (CRD) was conducted in a MLR-352H-PC illumination incubator (Panasonic, Kadoma, Japan) at the Guangxi Academy of Agriculture Sciences in Nanning. The Guangxi indica rice cultivar “Huanghuazhan” was chosen for its widespread use in southern China, displaying initial germination rates exceeding 95% and initial seed moisture content below 10% on a dry weight basis. The experiment consisted of three treatments: control (CK), 10 mg L^−1^ GA_3_, and 0.3 mg L^−1^ BR. These treatments were selected based on previous results by Zhang, Zhang, Gao, Ali, Wu, Tang, Chen, Jiang and Liang [1]. Initially the solutions were prepared, and healthy plump rice seeds were selected. The seeds were completely immersed in the initiation solution with a rice seed mass to initiation solution volume ratio of 1:5 (*w*/*v*) and initiated for 24 h in the dark at 25 °C. After initiation, the seeds were transferred into a mesh bag, rinsed with tap water for 3 min, drained, and dried in a 25 °C air-drying oven for 48 h to ensure that the total weight of the rice seeds remained the same as before treatment. Finally, the seeds were stored in a self-sealing bag in a 4 °C refrigerator for future experiments.

Each treatment involved a hundred seeds replicated three times, distributed onto individual 150 mL petri dishes layered with germination paper. The dishes were then transferred to an incubator, subjecting them to a day/night temperature of 12 °C/15 °C for chilling stress treatment. The light intensity was 12,000 lux, and the samples from embryo were collected at 0, 48, and 72 h.

### 2.2. Seed Germination Measurement and Sampling

Seed germination, defined as the embryo root breaking through the rice shell by 2 mm, was recorded every 24 h following chilling stress exposure. The seed germination rate was then calculated following the below formula.

$$(Germination\;rate)\;P_{G}=\frac{n}{N}\times 100\%$$

*P_G_* indicates germination rate, *n* is the number of normal germinated seeds, and *N* is the total number of tested seeds.

### 2.3. Total Protein Extraction

Total protein was extracted using a urea lysis buffer (8 M urea and 1% SDS) with a protease inhibitor. Using a high-throughput tissue grinder, each sample was shaken for 40 s and, three times following, dissolved on ice for 30 min with vortex mixing for 5–10 s every 5 min. The supernatant was collected after centrifugation (12,000× *g*, 4 °C for 30 min). For protein quantification, a Bradford protein assay kit was used. A BSA standard protein solution was prepared with concentrations ranging from 0 to 0.5 g/L. Each well of a 96-well plate was filled in triplicate with BSA standards and sample solutions at different dilutions, with each well containing 20 µL. To measure protein concentration, 180 µL of G250 dye was added to each well, followed by incubation for 5 min at room temperature. The absorbance at 595 nm was then measured to construct a standard curve for determining the protein content. For sodium dodecyl-sulfate polyacrylamide gel electrophoresis (SDS-PAGE) analysis, a 12% SDS-PAGE gel containing a 20 µg protein sample was prepared. The stacking gel was run at 80 V for 20 min, followed by the separating gel at 120 V for 90 min. Coomassie Brilliant Blue R-250 (Sigma-Aldrich, St. Louis, MO, USA)was used to stain the gel.

### 2.4. Protein Digestion and TMT Labeling

After 100 ug of protein was removed, TEAB was added to reach a final concentration of 100 mM. The reaction was then run for 60 min at 37 °C while TCEP (tris(2-carboxyethyl) phosphine) was added to reach the final concentration of 10 mM. After that, IAM (iodoacetamide) was added to bring the final concentration of IAM to 40 mM. The reaction was then allowed to proceed for 40 min at room temperature in a dark environment. After adding cooled acetone (sample volume = 6:1) to each tube, the mixture was precipitated at −20 °C for 4 h and then centrifuged at 10,000× *g* for 20 min. After completely dissolving the precipitate in 100 µL of 100 mM TEAB, trypsin was added at a mass ratio of 1:50 (protein: enzyme) to facilitate overnight enzymatic hydrolysis at 37 °C.

After being removed at −20 °C, the TMT reagent (No. A44522, Thermofisher, New York, NY, USA) was raised to room temperature. After adding acetonitrile, the mixture was vortexed. After labelling the samples CK, GA3, and BR, one tube of TMT reagent was added for every 100 μg of the polypeptide. The samples were then incubated at room temperature for two hours. After the addition, hydroxylamine was reacted for thirty minutes at room temperature. Identical amounts of the marked products were combined in a tube, and a vacuum concentrator was used to drain them. All of the samples were then combined and vacuum-dried.

### 2.5. High pH RPLC Separation

To boost proteome depth, we fractionated materials using high-pH reverse phase separation. After being re-solubilized with UPLC loading buffer (2% acetonitrile (ammonia to pH 10)), the peptide samples were separated in a high-pH liquid phase using an ACQUITY UPLC BEH C18 Column 1.7 µm, 2.1 mm × 150 mm reversed-phase C18 column (Waters, Milford, MA, USA). Over the course of 48 min, at a flowrate of 200 μL/min, peptides were separated using a gradient of elution (Phase A: 2% acetonitrile, pH 10; Phase B: 80% acetonitrile, pH 10). The gradient used to elute the peptides was as follows: 0–1.9 min 0–0% B; 1.9–2 min 0–5% B; 2–17 min 5–5% B; 17–18 min 5–10% B; 18–35.5 min 10–30% B; 35.5–38 min 30–36% B; 38–39 min 36–42% B; 39–40 min 42–100% B; 40–44 min 100% B; 44–45 min 100~0% B; 45–48 min 0% B. Following the collection of 28 fractions based on peak form and timing, 14 fractions were merged and vacuum centrifuged to concentrate the mixture.

### 2.6. LC-MS/MS Analysis

According to the usual protocols of Majorbio Bio-Pharm Technology Co. Ltd. (Shanghai, China), two-dimensional analysis was carried out by liquid chromatography tandem mass spectrometry (Evosep One paired with Obitrap Exploris 480 mass spectrometer (Thermo Fisher Scientific, Bremen, Germany)). For liquid phase separation, the peptide mixture was placed onto a C18 column (150 μm × 15 cm, Evosep, Odense, Denmark) that was subjected to a linear gradient of solvent B (100% ACN with 0.1% formic acid) at a flow rate of 300 nL/min. Solvent A was water with 0.1% formic acid.

The data-dependent acquisition mode (DDA) of the Orbitrap Exploris 480 was used to automatically transition between full scan MS and MS/MS acquisition. The Orbitrap was used to obtain the full scan MS spectra (*m*/*z* 350–1500) at 60 K resolution. After that, precursor ions were chosen and added to the collision cell so that higher-energy collision dissociation (HCD) could fragment them. Dynamic exclusion was set to 30 s, and the MS/MS resolution was fixed at 15 K.

### 2.7. Identification of Proteins

Proteome Discoverer (Thermo Scientific, Version 2.2 (Waltham, MA, USA)) was used to analyze the RAW data files against the UniProt (UniProt Consortium, 2019) database. The fragment mass tolerance was fixed at 0.02 Da, and the precursor mass tolerance at 20 ppm. FDR < 0.01 was established as the false discovery rate (FDR) for peptide identification. To assist protein identification, at least one distinct peptide identification was employed.

### 2.8. RNA Extraction and Quantitative Real-Time Transcription Polymerase Chain Reaction (qRT-PCR)

Total RNAs were extracted from each root treatment and control separately, according to the manufacturer’s protocol for the TRIzol Reagent (Invitrogen, Shanghai, China). The extracted RNAs were treated with RNase-free DNase 1 (Takara, Dalian, China) to eliminate the DNA residues. Extracted RNA was reverse-transcribed into cDNA using MMLV First Strand cDNA Synthesis Kit (Invitrogen, Shanghai, China), and 400 ng RNA was used for each sample. The validation of the proteomics data through gene expression was carried out by q RT-PCR, and proteins were chosen for verification. Specific PCR primers of the selected genes were designed using Primer3 [23]. Gene expression was normalized using ubiquitin as an internal control, and primers are listed in Supplementary File—Table S1. Samples and standards were performed on a CFX96 Real-Time PCR System (Bio-Rad, Hercules, CA, USA) using the SYBR Green Super Mix kit (Bio-Rad, Hercules, CA, USA). Each sample and standard was run in triplicate on each plate. Each Real-Time PCR was performed in a 20 μL reaction volume containing 10 μL SYBR Green Super Mix, 7 μL ddH2O, 1 μL forward primer (10 μmol/L), 1 μL reverse primer (10 μmol/L), and 1 μL template cDNA. The PCR programs were run as follows: 5 min of pre-denaturation at 95 °C, 39 cycles of 15 s at 95 °C, 30 s at 55 °C, and 15 s at 72 °C. The transcription abundance of selected genes was calculated by 2^−△△Ct^ comparative threshold cycle (Ct) method and normalized with the results of 18 s gene [24].

### 2.9. Statistical Analysis

Seed germination rate and genes expression data underwent one way analysis of variance (ANOVA) using the Statistix 8.1 software (Analytical Software, Tallahassee, FL, USA). The least significant difference test was used to compare the treatment means at *p* ≤ 0.05. The Wukong Cloud Platform, which is available for free online, was used to analyze the data (www.omicsolution.com, accessed on 5 June 2023). *p*-value ≤ 0.05 and fold change (≥2 or ≤0.5) criteria were utilised to find DEPs. Using the GO (http://geneontology.org, accessed on 14 June 2023) and KEGG pathway (http://www.genome.jp/kegg/, accessed on 17 June 2023) tools, all discovered proteins were annotated. DEPs were further employed in KEGG and GO enrichment analyses. The analysis of protein–protein interactions was conducted with String v10.5. For functional annotation, the Blast2GO software (http://www.blast2go.com/, accessed on 19 June 2023), an automated tool for assigning GO terms, conducted Gene Ontology (GO) annotation analysis.

## 3. Results

### 3.1. The Effect of Different Initiators on the Germination Rate of Rice Seeds under Chilling Stress

**Figure 1:**
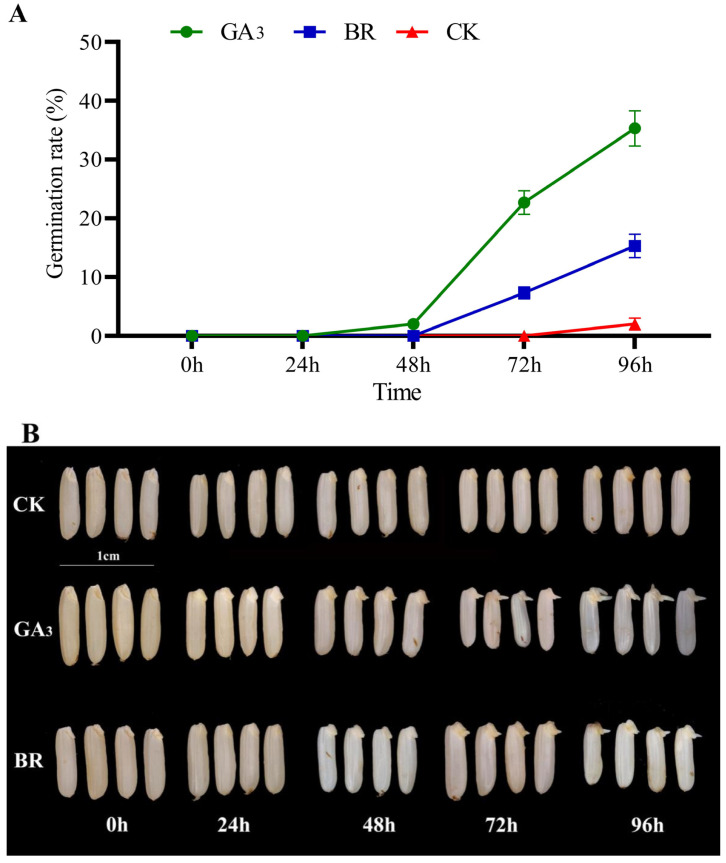
(**A**) The effect of different initiators on the germination rate of rice seeds under low-temperature stress. (**B**) Rice seed germination phenotypes under different treatments (*p* ≤ 0.05). Note: The seeds were completely immersed in the GA_3_ (10 mg L^−1^ gibberellin A3), BR (0.3 mg L^−1^ brassinolide) and CK (water as control) to initiate for 24 h in the dark at 25 °C and dry. They were subjected to a day/night temperature of 12 °C/15 °C for chilling stress treatment. Error bars represent the stand deviation. Scale bar = 1 cm.

Seed priming with GA_3_ and BR significantly impacted rice germination under low-temperature stress (Figure 1A,B). Compared to the control (CK), GA_3_ and BR exhibited the highest rates at 72 h (22.67% and 7.33%, respectively) and at 96 h (35% and 15%, respectively). GA_3_ treatments showed a significantly higher germination rate than BR among the priming treatments. Figure 1B illustrates clear embryo emergence in all treatments.

### 3.2. Protein Detection

**Figure 2:**
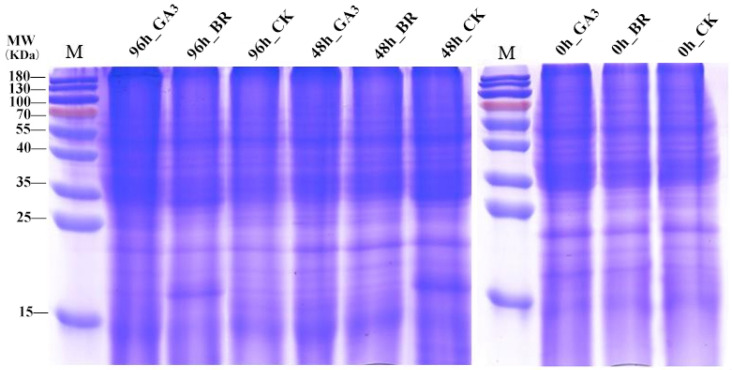
SDS-PAGE gel electrophoresis. Note: The numbers in the figure represent different treatments, and M represents standard proteins.

To better explore how initiators affect seed germination under low-temperature stress, this study conducted proteomic analysis using mass spectrometry on rice embryos treated with GA_3_ and BR initiators (Figure 2). Following protein extraction from the rice seed embryos, a thorough protein quality evaluation was conducted. SDS-PAGE, Sodium dodecyl sulfate polyacrylamide gel electrophoresis, is a commonly used technique to separate proteins and peptides from samples according to their molecular weight. SDS-PAGE gel electrophoresis technology was utilized to ensure clear band definition in the samples, with no evidence of tailing or protein degradation observed. These results confirm that the sample quality meets the necessary standards for subsequent enzymatic hydrolysis, TMT labeling, and mass spectrometry analysis.

### 3.3. Protein Mass Spectrometry Identification

**Figure 3:**
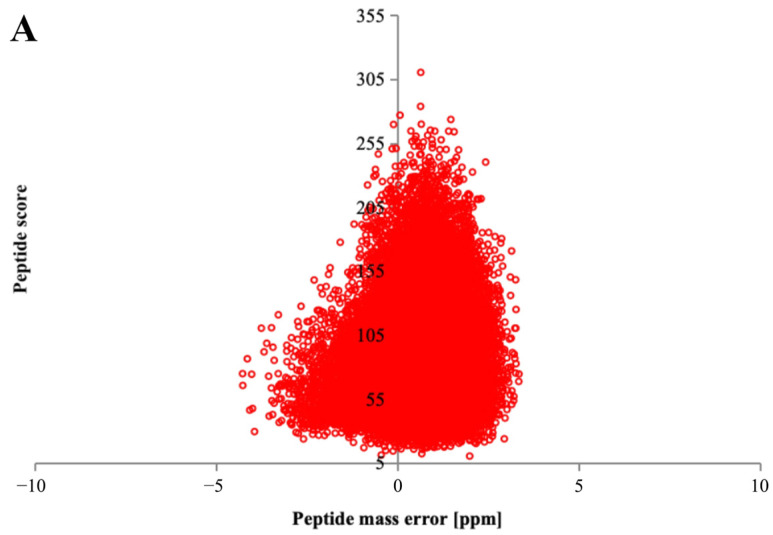

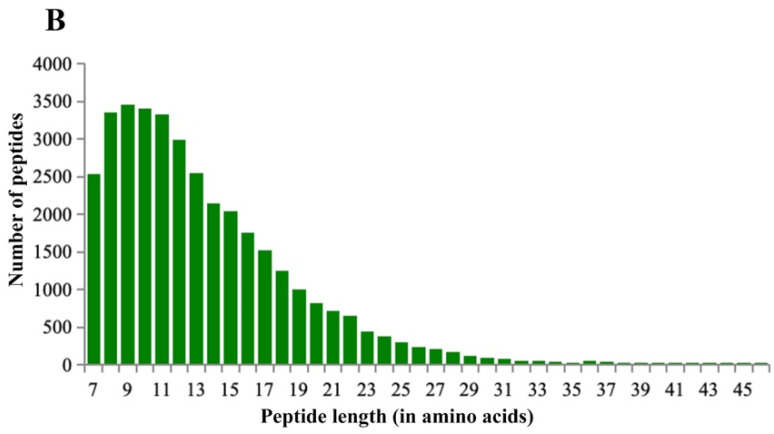
Quality control detection results of mass spectrometry data. Note: (**A**) Identify the mass shift distribution of peptide segments, and (**B**) determine the length distribution of peptide segments.

Quality control analysis was conducted on the mass spectrometry detection results, which showed that the mass error of the peptide segment in the mass spectrometry data was less than 10 ppm (Figure 3A), indicating that the quality error met the requirements. Moreover, the distribution of peptide segments predominantly falls within 8–20 amino acid residues (Figure 3B), aligning with the expected pattern of pancreatic enzyme digestion of peptide segments. This indicates that the sample preparation meets the necessary standards.

### 3.4. Identification of Rice Embryo Proteins Induced by GA_3_, BR, and CK

**Figure 4:**
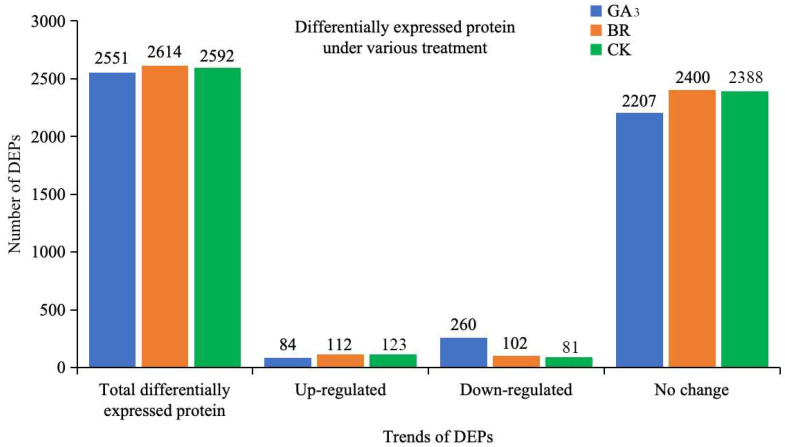
Proteins obtained from rice embryos under the GA_3_, BR, and CK treatments. Note: The *X*-axis indicates the trend of proteins, and the *Y*-axis shows the numbers of proteins per trend type. GA_3_: 10 mg L^−1^ gibberellin A3, BR: 0.3 mg L^−1^ brassinolide and CK: water as control.

Differential analysis was conducted on the LC-MS/MS proteomic data of GA_3_ and BR-induced embryos with TMT quantitative markers, as well as on the control, using a multitude of differences with FC ≥ 2 and *p* ≤ 0.05 as cutoff values (Figure 4). The results of the differential analysis revealed that, for rice embryos treated with GA_3_ compared to those without germination, a total of 2551 proteins were identified during the germination process from 48 to 96 h. Among these, 84 proteins were significantly upregulated, and 260 were downregulated. Similarly, for rice embryos treated with BR compared to before germination, a total of 2614 proteins were identified during seed germination from 48 to 96 h. Among these, 112 proteins were significantly upregulated, and 102 were downregulated. Moreover, CK treatment showed 2592 proteins, of which 123 proteins were significantly upregulated and 81 proteins were downregulated (Figure 4).

### 3.5. Fuzzy Clustering and COG Functional Classification of Differentially Expressed Proteins in Rice Embryos Induced by GA_3_, BR, and CK

**Figure 5:**
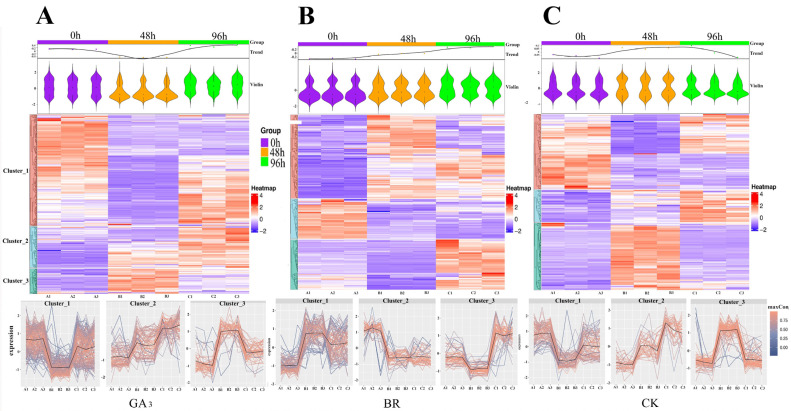
Fuzzy clustering of DEPs induced by (**A**) GA_3_, (**B**) BR, and (**C**) CK. Note: All the proteins were sorted into three clusters based on their expression patterns.

To investigate the expression patterns, potential functions, and roles of proteins at various stages throughout the dynamic process of seed germination, we conducted fuzzy clustering and COG analysis on proteins from different treatment groups. The results from Mfuzzy revealed distinct patterns for differentially expressed proteins (DEPs) induced by GA_3_. These DEPs were categorized into three clusters: Cluster 1 comprised 215 DEPs that were upregulated at 0 h, downregulated at 48 h, and then upregulated again at 96 h. Cluster 2 consisted of 80 DEPs showing a gradual upregulation over time, while Cluster 3 included 49 DEPs upregulated at 48 h and subsequently downregulated at 96 h. Similarly, DEPs induced by BR were classified into three clusters: Cluster 1 contained 103 DEPs downregulated at 0 h, followed by upregulation at 48 and 96 h. Cluster 2 encompassed 49 DEPs upregulated at 0 h but downregulated at 48 and 96 h. Cluster 3 involved 62 DEPs downregulated at 0 and 48 h but upregulated at 96 h. Moreover, DEPs induced by CK were also divided into three clusters: Cluster 1 featured 86 DEPs downregulated at 0 h and then upregulated at 48 and 96 h. Cluster 2 included 37 DEPs upregulated at 0 h but downregulated at 48 and 96 h. Cluster 3 consisted of 77 DEPs downregulated at 0 and 48 h but upregulated at 96 h (Figure 5A).

**Figure 6:**
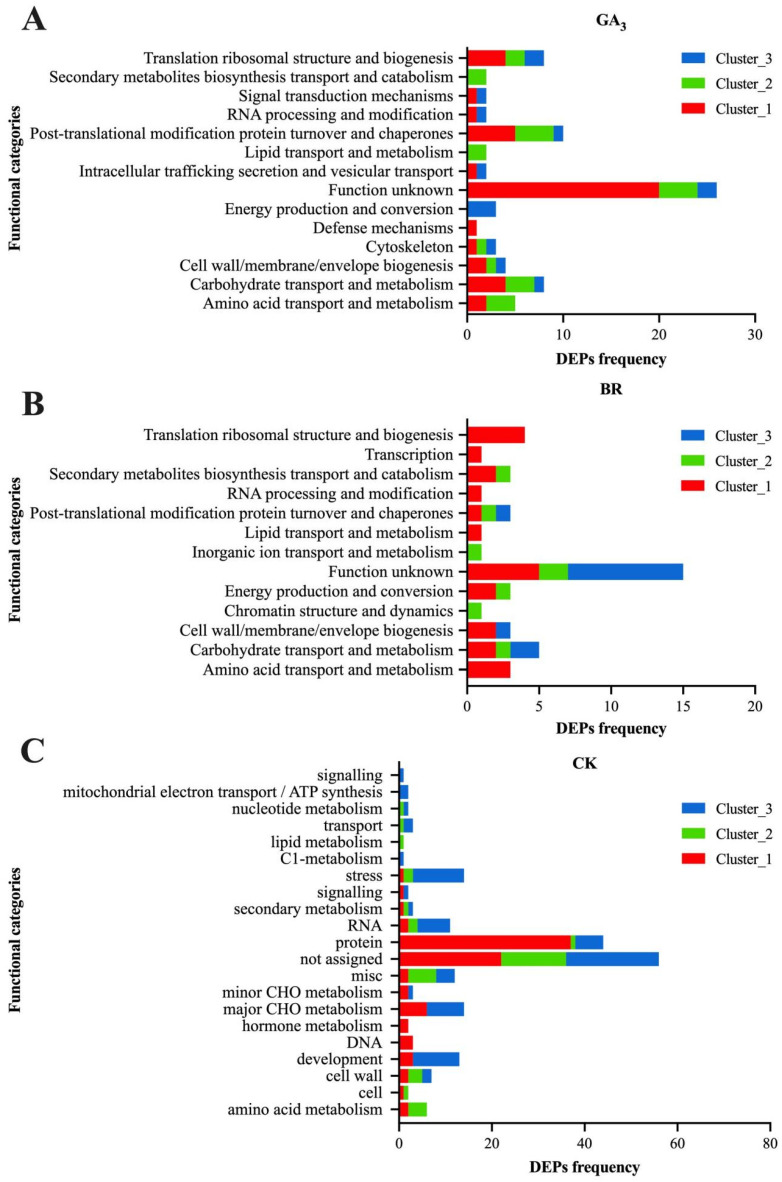
COG functional classification of DEPs induced by (**A**) GA_3_, (**B**) BR, and (**C**) CK. Note: (**A**–**C**) GA_3_, BR, and CK induce the COG function of DEPs in rice embryos. The *x*-axis represents the functional types of DEPs, while the *y*-axis represents the number of DEPs in each functional type. Clusters 1, 2, and 3 represent the replications.

To further classify DEPs based on functional annotations, COG analysis was performed on the aforementioned protein clusters (Figure 6A–C). For GA_3_ treatment, a total of 42, 24, and 15 DEPs were identified across three clusters, each associated with 11, 9, and 10 functional categories, respectively. Excluding the functions of unknown proteins, in clusters 1 and 2, most proteins were related to post-translational modifications, protein transport, carbohydrate metabolism, translation, ribosome structure, and biosynthesis. In cluster 3, the proteins were mainly concentrated in energy generation and transport. Similarly, for BR treatment, 24, 8, and 12 DEPs were identified across three clusters, each associated with 11, 7, and 5 functional categories, respectively. In cluster 1, the majority of proteins were involved in biosynthesis, translation, ribosomal structure, and secondary metabolite biosynthesis. Cluster 2 proteins were mainly associated with post-translational modifications, protein turnover, and chaperone proteins, while cluster 3 proteins were primarily involved in carbohydrate transport and metabolism. For CK treatment, 87, 37, and 78 DEPs were divided into 21, 12, and 15 functional categories, respectively. In cluster 1, most proteins were related to protein categories, while cluster 2 proteins were mainly concentrated in amino acid metabolism functions. Cluster 3 proteins were primarily focused on stress resistance functions.

### 3.6. GO Analysis of DEPs in Rice Embryos Induced by GA_3_, BR, and CK

In addition to fuzzy clustering analysis, GO (Gene Ontology) clustering analysis was conducted on proteins with continuously upregulated and downregulated expression multiples, with the aim of combining the two methods to further explore the biological correlation of initiators in promoting seed germination dynamics.

**Figure 7:**
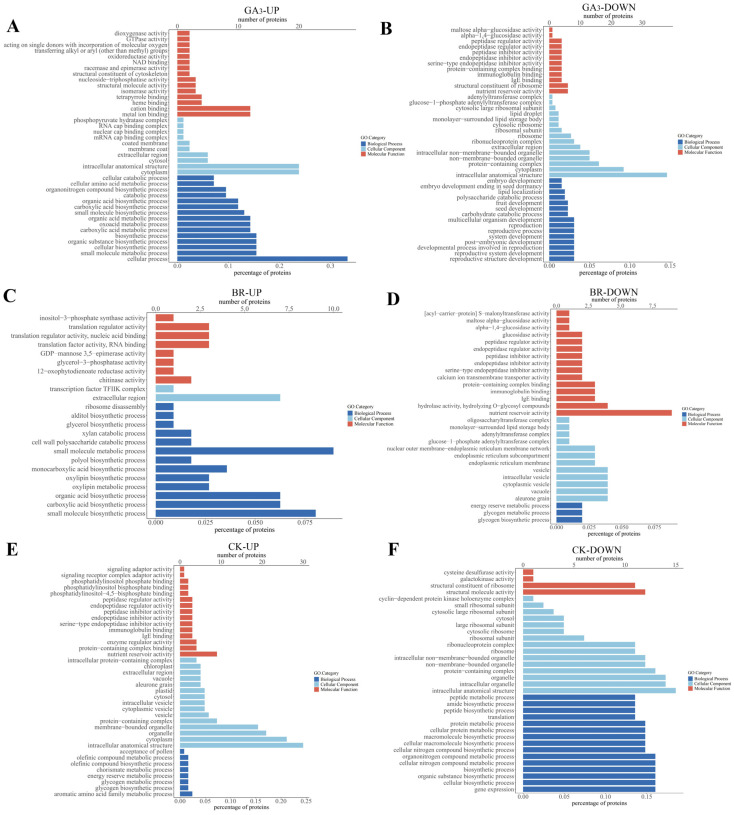
In GA_3_, BR and CK functional analysis of GO of DEPs in the rice embryo. Note: the *x*-axis represents the GO functional categories (biological processes, cell components, and molecular functions) of differential proteins, and the *y*-axis represents the number of DEPs in each functional category (*p*-value ≤ 0.05). (**A**) GA_3_ up-egulaed DEPs; (**B**) GA_3_ download regulaed DEPs; (**C**) BR up-regulaed DEPs; (**D**) BR down regulaed DEPs; (**E**) CK up-regulaed DEPs; (**F**) CK down regulaed DEPs.

The GO enrichment analysis results revealed that 172 upregulated differential proteins were primarily involved in biological processes such as cellular processes and small molecule metabolism in rice embryos induced by GA_3_. These proteins were distributed within cellular positions such as the cytoplasm and intracellular anatomical structures. Their molecular functions mainly revolved around ion binding (Figure 7A). Conversely, 54 downregulated DEPs were mainly associated with biological processes such as reproduction (3.08%) and multicellular biological development (3.08%). They were distributed in anatomical structures (14.62%) and the cytoplasm (9.23%) within the cell. Their molecular functions included nutrient bank activity, ribosome structural components, and IgE antibodies (Figure 7B).

After BR-induced rice embryos, 23 upregulated differential proteins were found to primarily participate in biological processes such as small molecule biosynthesis (8.04%), small molecule metabolism (8.93%), and organic acid biosynthesis (6.25%). These proteins were distributed in the extracellular space (6.25%) and other locations. Their molecular functions mainly centered around chitinase activity (1.79%) (Figure 7C). Conversely, 37 downregulated DEPs were primarily involved in biological processes such as glycogen biosynthesis (1.96%), glycogen metabolism (1.96%), and energy reserve metabolism (1.96%). They were distributed in positions such as aleurone granules (3.92%) and cytoplasmic vesicles (3.92%). Their molecular functions included nutrient pool activity (8.82%) and hydrolytic enzyme activity (3.92%) (Figure 7D).

### 3.7. KEGG Analysis of DEPs in Rice Embryos Induced by GA_3_, BR, and CK

**Figure 8:**
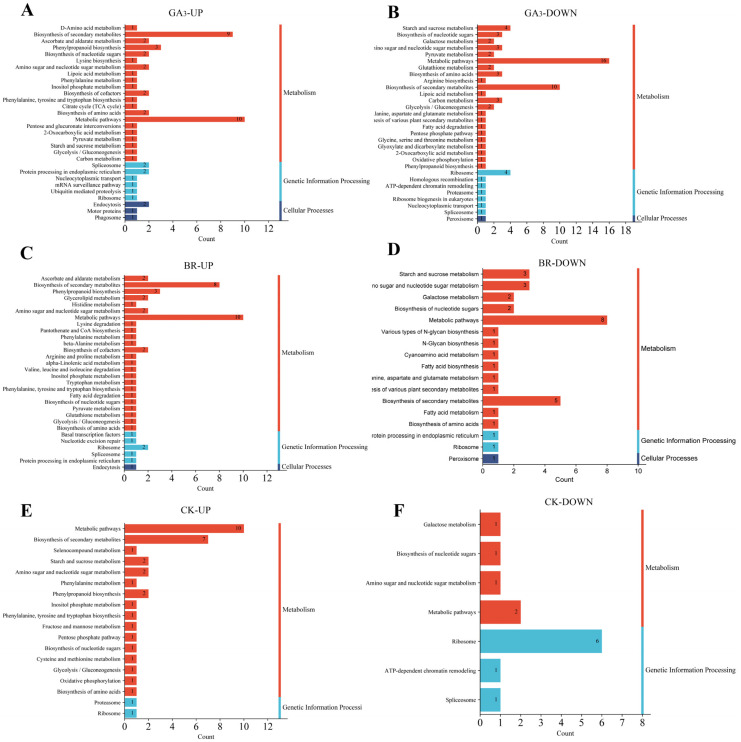
GA_3_, BR, and CK induced the KEGG function of DEPs in rice embryos. Note: The *x*-axis represents the KEGG functional classes of DEPs (metabolism, genetic information processing, environmental information processing, cellular processes, and biological systems), while the *y*-axis represents the number of DEPs in each functional class. (**A**) GA_3_ up-egulaed DEPs; (**B**) GA_3_ download regulaed DEPs; (**C**) BR up-regulaed DEPs; (**D**) BR down regulaed DEPs; (**E**) CK up-regulaed DEPs; (**F**) CK down regulaed DEPs.

To further explore the potential signaling pathways involved in promoting low-temperature seed germination, this study collected DEPs from rice embryos induced by GA_3_, BR, and CK at 0, 48, and 96 h, respectively. The obtained DEPs were subjected to pathway enrichment analysis based on the Kyoto Encyclopedia of Genes and Genomes (KEGG), and functional annotations were obtained (Figure 8). This study found that the differential proteins in rice embryo identification induced by GA_3_, BR, and CK were mainly expressed in three pathways: metabolism, genetic information processing, and cellular processes.

The upregulated proteins in rice embryos induced by GA_3_ mainly participate in secondary pathways such as metabolic pathways (10), the biosynthesis of secondary metabolites (9), phenylpropanoid biosynthesis (3), splicing bodies (2) during gene information processes, and protein processing in the endoplasmic reticulum (2). Additionally, involvement is observed in endocytosis (2), motor proteins (1), and phagosomes (1) during cellular processes.

On the other hand, the main secondary pathways involved in downregulating proteins include metabolic pathways (16) and the biosynthesis of secondary metabolites (10), as well as starch and sucrose metabolism (4), amino acid biosynthesis (3), and carbon metabolism (3). Furthermore, ribosomes (4) are affected in genetic information processes, and peroxisomes (1) in cellular processes.

After BR induction, both upregulated and downregulated proteins in rice embryos are involved in metabolic pathways (10, 8) and the biosynthesis of secondary metabolites (85). Additionally, upregulated proteins are also involved in phenylpropanoid biosynthesis (3) and cofactor biosynthesis (2), as well as ribosome activity (2) during genetic information processes and endocytosis (1) during cellular processes. The downregulation of proteins affects starch and sucrose metabolism (3), as well as amino and nucleotide sugar metabolism (3). Furthermore, there is an impact on protein processing in the endoplasmic reticulum and ribosomes in genetic information (1), as well as peroxisomes in cellular processes (1).

The main secondary pathways identified in upregulated proteins in rice embryos treated with CK control include metabolic pathways (10) and the biosynthesis of secondary metabolites (7). Additionally, there is involvement in proteasomes (1) and ribosomes (1) in genetic information processes. On the other hand, downregulated proteins are associated with galactose metabolism (1), nucleotide biosynthesis (1), and amino and nucleotide metabolism (2). There is also an impact on ribosome activity (6), ATP-dependent chromatin remodeling (1), and spliceosomes (1) in gene information processes.

The analysis of differential protein pathway enrichment in embryos induced by GA_3_ under low-temperature stress at 48 and 96 h of germination revealed notable findings. A total of 23 DEPs were enriched in the pathway, with 11 upregulated at both 48 and 96 h, and nine downregulated at the same time points (Table 1). Notably, the mRNA splicing factor (Q5Z414) exhibited the highest upregulation (FC = 17.032) at 48 h, while phenylalanine ammonia lyase (P14717) showed the highest upregulation (FC = 12.649) at 96 h.

In the samples treated with BR, a total of 16 DEPs were enriched in the pathways based on differential protein pathway enrichment analysis between 48 h and 0 h, as well as between 96 h and 0 h. Among these, seven DEPs were upregulated after 48 h and 96 h of treatment, while nine DEPs were downregulated at the same time points. Notably, Inositol-3-phosphate synthase (O64437) and phenylalanine ammonia lyase (P14717) were shown to be significantly upregulated after 48 h of treatment, with FC values reaching 7.191 and 6.882, respectively. Subsequently, after 98 h of treatment, these values increased to 7.754 and 9.259, respectively (Table 2).

In the samples without seed priming (CK), differential protein pathway enrichment analysis between 48 h and 0 h, as well as between 96 h and 0 h, revealed the significant enrichment of a total of 13 DEPs in the pathway. Among these, seven DEPs were upregulated under both 48 and 96 h of treatment, while six DEPs were downregulated at the same time points. Notably, at 48 and 96 h of treatment, Ent sandaracopimaradiene 3-hydroxylase (Q0DBF4) and potential calmodulin (Q7XHW4) exhibited significant upregulation (Table 3).

### 3.8. Expression Patterns of Genes Related to Observed Proteins

**Figure 9:**
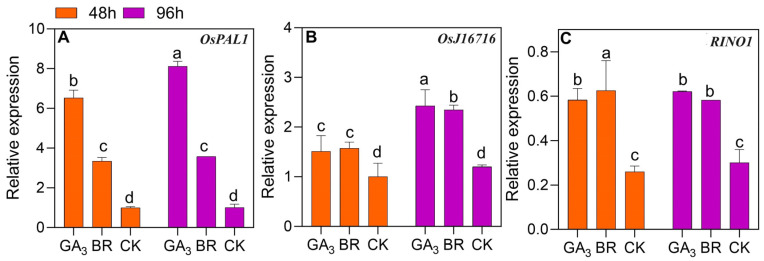
Relative gene expression under cold stress in 48 and 96 hours’ embryo during seedlings of rice; mRNAs extracted from the embryos were analyzed using qRT-PCR. Three proteins that exist in all three treatments (GA_3_, BR, and CK) were selected. Different letters above the columns indicate significant differences among all the seed priming treatments during both timings. (**A**) expression of *OsPAL1* gene; (**B**) expression of *OsJ_16716* gene; (**C**) expression of *RINO1* gene.

The expression level of genes encoding Phenylalanine ammonia-lyase, Peroxidase, and Inositol-3-phosphate synthase-related proteins were determined to display the impact of GA_3_ and BR treatment on rice tolerance under cold stress (Figure 9). The relative expression levels of *OsPAL1* (*Os02g0626100*), *RINO1* (*Os03g0192700*), and *OsJ_16716* (*Os04g0688200*) were upregulated at both 48h and 96h, and the expression levels increased with time. Compared with the control, these genes had higher expression levels in GA_3_ and BR treatments. Overall, the expression patterns of these genes are implicated in complex processes during rice germination under cold stress.

## 4. Discussion

### 4.1. Seed Germination

The initial spring’s low temperature poses a significant environmental threat to rice seedlings, hindering growth and agricultural productivity [25,26]. Cold stress not only injures and kills plants but also hampers their growth and development, ultimately leading to reduced crop yield. In the present study GA_3_ exhibited the highest rates of germination compared to control at 96h, followed by BR treatment (Figure 1). These results can be attributed to the fact that GA_3_ is known as a growth promoter hormone that inhibits water uptake accompanying embryonic growth [27]. Furthermore, GA_3_ plays key roles in the physiological and metabolic processes that trigger seed germination [28] and helps mitigate the adverse effects of stress. Furthermore, higher germination in BR can be attributed to its vital role in regulating plant growth and development, including cell elongation, seed germination, microtubule arrangement, cell division, and differentiation [29].

### 4.2. Effects of GA_3_ and BR Seed Initiation on Protein Content in Low-Temperature Germinated Rice Embryos

Gibberellin A3 (GA_3_) plays a crucial role in plant growth resistance to low-temperature stress [30], by binding cell membrane receptors, breaking down and activating GA-MYB transcription factors to promote seed germination [31,32]. BR regulates plant cell division, elongation, and gene expression, improving plant responses to abiotic stresses such as heat, cold, drought, salt, pesticides, and heavy metals [33]. BR can stimulate ethylene synthesis, membrane hyperpolarization, and sucrase activity, promoting osmotic regulation, and provide energy for plant growth [33,34]. This study shows that GA_3_ and BR influence peroxide metabolism, phenylpropanoid biosynthesis, and inositol phosphate metabolism. Among them, peroxidase (A3AYV8 and A0A0N7KM90) plays an important role in removing H_2_O_2_ accumulation caused by stress, indicating that triggering treatment promotes the expression of peroxidase activity under low-temperature stress. Phenylalanine ammonia lyase (P14717) plays an important role in plant physiological metabolism and stress response [35]. Chun-juan et al. [36] found that spraying specific inhibitors increased its activity in cucumber seedlings, enhancing their cold resistance. Our results showed that GA_3_ and BR upregulated P14717 protein, increasing phenylalanine ammonia lyase activity, phenylpropanoid synthesis, secondary metabolites, and antioxidant enzyme, which alleviate oxidative damage caused by low temperature. In addition, RayChaudhuri et al. [37] found that under salt stress, the activity of inositol-3-phosphate synthase in rice chloroplasts increased under photosensitivity, indicating that this enzyme has a promoting effect on plant resistance to stress. Similar to this, our study also found that GA_3_ and BR upregulated inositol-3-phosphate synthase seed embryos, with expression peaking at 96 h. This suggests enhanced phytic acid biosynthesis, providing nutrients for better seed germination and stress resistance.

In addition, GA_3_ also stresses responsive protein activation, GTP activation, and ascorbic acid biosynthesis. Among them, heat shock protein, initially linked to high-temperature stress, is also involved in the response of plants to non-biological stresses such as low temperature [38]. In this study, the heat shock homolog 70 kDa protein 2 (Q84TA1) was upregulated, indicating that GA_3_ can maintain the cell membrane’s stability, support resistance to low temperature damage, and participate in the antioxidant response and hormone regulation of signal transduction in plant cells, thereby increasing seed stress resistance [39,40]. The upregulation of ARF GAP like zinc finger protein ZIGA2 (Q0DLN9) enhances plant growth, development, and resistance mechanisms in seeds. Similar to our research findings, Abdirad et al. [41] found that under drought stress conditions, ARF GAP like zinc finger protein was upregulated in rice. In addition, the upregulation of GDP mannose 3,5-dimerase (A3C4S4) expression is crucial for mannose synthesis, suggesting that GA_3_ triggers GDP-D-mannose isomerization, promoting vitamin C (L-ascorbic acid) biosynthesis [42,43]. At the same time, it also promotes cell wall biosynthesis by increasing polysaccharide content, increasing the hardness and adhesion ability of the cell walls, and adsorbing and killing pathogens, thereby improving stress resistance [44,45,46].

Meanwhile, BR triggers specific involvement in ribosome synthesis and amino acid synthesis, regulating the upregulation of 60S ribosomal protein (B7F845) and phospho-2-dehydro-3-deoxyheptanate aldolase (Q75W16) expression. The 60S ribosomal protein, a component of the large subunit in plant cells responsible for protein synthesis, plays a crucial role under low-temperature stress by participating in the biosynthesis of 60S ribosomal subunits, thereby regulating precursor rRNA processing and ribosome synthesis. Ribosomes serve as key regulatory nodes in response to cold stress, modulating the synthesis of protective proteins like antifreeze proteins and heat shock proteins. The 60S ribosomal protein regulates protective protein synthesis [47], enabling plants to maintain growth rate under low-temperature conditions and reduce the negative impact of stress [48]. Previous studies show its role in enhancing drought and salt tolerance in cotton [49,50]. In this study, BR induction upregulated 60S ribosomal protein, boosting ribosomal activity and protein synthesis, which accelerates the production of essential proteins for cold resistance stress [51,52]. Additionally, BR upregulates phospho-2-dehydro-3-deoxyheptanate aldolase, which catalyzes amino acid biosynthesis and regulates plant response to stress through endogenous hormones and signaling pathways, thereby enhancing plant stress resistance [53].

### 4.3. Genes Expression Patterns

The three genes common in all treatments were selected to evaluate the response of rice tolerance to cold stress under different seed priming treatments (Figure 9). Among them, *OsPAL1* was highly expressed in GA_3_ treatment, particularly at 96h. This gene is directly involved in salicylic acid (SA) synthesis, which is crucial for enhancing stress resilience [54]. Supporting our results, a study found that an increase in SA synthesis improved plant’s resilience against low-temperature stress during germination. Furthermore, our study also confirmed that the *OsPAL1* gene is involved in protein such as phenylalanine ammonia-lyase proteins, which were also upregulated under GA_3_ treatments (Table 1). Moreover, the strong upregulation of the *OsJ_16716* gene in GA_3_ and BR at 96 h highlights the key role of peroxidase (A3AYV8 and A0A0N7KM90) in reducing stress-induced H_2_O_2_ accumulation and enhancing stress tolerance.

The expression of the *inositol-3-phosphate synthase 1-like (RINO1)* gene was demonstrated higher in both GA and BR compared to CK (Figure 9C). This gene catalyzes the conversion of glucose 6-phosphate to 1-inositol-1-phosphate, participating in inositol synthesis and phytic acid synthesis [37]. This indicated that both GA_3_ and BR increased the biosynthesis of phytic acid, providing phosphorus, mineral nutrients, and inositol for seed germination, leading to enhanced seed germination ability under cold stress. Moreover, the upregulation of Inositol-3-phosphate synthase (O64437) protein in both GA_3_ and BR also confirmed the enhanced biosynthesis of phytic acid, thus underscoring their role in bolstering seed germination ability under cold stress and paving the way for robust seedling development.

## 5. Conclusions

GA_3_ and BR exhibit both similarities and differences in their regulation of metabolism. Under low-temperature stress, GA_3_ and BR jointly participate in peroxide metabolism, phenylpropanoid biosynthesis, and inositol phosphate metabolism, enhancing antioxidant capacity and providing energy substances for germination. However, GA_3_ predominantly upregulates heat shock proteins and GDP mannose 3,5-dimerase, preserving cell stability and minimizing damage, while BR activates 60S ribosomal proteins and phospho-2-dehydro-3-deoxyheptanate aldolase, improving biosynthesis ability and metabolic regulation to sustain plant activities under low temperature stress. Furthermore, the heightened expression of genes (*OsJ_16716*, *OsPAL1*, *RINO1*) confirmed that GA^3^ boosts salicylic acid synthesis, enhancing resilience, whereas BR activates genes crucial for biosynthesis and metabolic regulation, aiding seed germination. Both treatments also enhance phytic acid biosynthesis, fortifying seed germination under cold stress.

## Figures and Tables

**Table 1:** KEGG enrichment analysis of DEPs obtained during GA_3_ treatment for 48 and 98 h.

Gene Names	Protein Accession	Description	Fold Change (FC)		Regulation
			48/0 h	96/0 h	
Os03g0821100	Q84TA1	Heat shock cognate 70 kDa protein 2	2.878	2.881	UP
Os04g0688200	A3AYV8	Peroxidase	4.537	6.489	UP
Os03g0854100	Q0DLN9	Similar to ARF GAP-like zinc finger-containing protein ZIGA2	3.618	3.641	UP
Os02g0612300	Q84L14	Nuclear cap-binding protein subunit 2	3.734	4.930	UP
Os03g0192700	O64437	Myo-inositol 3-phosphate synthase 1	7.521	8.943	UP
Os02g0739600	Q6Z5N4	Pyruvate dehydrogenase E1 component subunit	6.098	4.130	UP
Os10g0417600	A3C4S4	GDP-mannose 3,5-epimerase 1	6.235	6.112	UP
Os12g0567200	Q2QNF7	Diaminopimelate epimerase	6.682	4.608	UP
Os03g0331700	Q10LX4	Probable calcium-binding protein	8.361	7.654	UP
Os02g0626100	P14717	Phenylalanine ammonia-lyase	8.451	12.649	UP
Os06g0730800	Q5Z414	mRNA splicing factor	17.032	0.010	UP
Os07g0693100	Q0D3D2	Pyruvate decarboxylase	0.010	0.010	Down
Os01g0231700	Q5NB69	Elongation step of protein synthesis.	0.010	5.696	Down
Os01g0374000	Q93WM2	Glutathione transferase	0.133	0.170	Down
Os06g0219600	B9FS82	Similar to Poly(A)-binding protein II-like.	0.156	4.831	Down
Os01g0500900	A0A0P0V2Y9	Tyrosine--tRNA ligase	0.211	0.235	Down
Os05g0595100	Q8LNZ3	UDP-glucose 4-epimerase	0.238	0.144	Down
Os06g0675700	Q653V7	Alpha-glucosidase in rice seeds	0.29	0.397	Down
Os07g0184300	P31674	40S ribosomal protein S15	0.339	0.219	Down
Os07g0675100	Q6ZDX2	Pectinesterase	0.400	0.010	Down

**Table 2:** KEGG enrichment analysis of DEPs obtained during BR treatment for 48 and 98 h.

Gene Names	Protein Accession	Description	Fold Change (FC)		Regulation
			48/0 h	96/0 h	
Os04g0688200	A3AYV8	Peroxidase	4.624	5.788	UP
Os08g0558800	B7F845	60S ribosomal protein	2.476	3.373	UP
Os03g0192700	O64437	Inositol-3-phosphate synthase	7.191	7.754	UP
Os02g0626100	P14717	Phenylalanine ammonia-lyase	6.882	9.259	UP
Os07g0622200	Q75W16	Phospho-2-dehydro-3-deoxyheptonate aldolase	5.189	4.498	UP
Os10g0517500	Q7XCS3	Cys/Met metabolism PLP-dependent enzyme family protein	2.799	3.545	UP
Os07g0681400	Q7XHW4	Probable calcium-binding protein	3.629	3.103	UP
Os02g0169900	Q6H6B9	Inositol-1-monophosphatase	0.010	0.010	Down
Os06g0708832	Q5Z9H5	Similar to arogenate dehydrogenase.	0.010	0.010	Down
Os02g0738900	Q0DXR0	Dynamin GTPase	0.135	0.179	Down
Os05g0595100	Q8LNZ3	UDP-glucose 4-epimerase	0.190	0.186	Down
Os01g0633100	Q7G065	Glucose-1-phosphate adenylyltransferase large subunit	0.232	6.741	Down
Os06g0320200	Q5Z9Z0	Beta-glucosidase	0.245	0.028	Down
Os06g0172600	Q69SI5	40S ribosomal protein	0.386	0.020	Down
Os05g0461400	Q6L500	Probable histone H2A.	0.492	0.021	Down

**Table 3:** KEGG enrichment analysis of DEPs obtained during CK treatment for 48 and 98 h.

Gene Names	Protein Accession	Description	Fold Change (FC)		Regulation
			48/0 h	96/0 h	
Os04g0688200	A3AYV8	Peroxidase	2.530	4.491	UP
Os10g0517500	Q7XCS3	Cys/Met metabolism PLP-dependent enzyme family protein	3.420	6.565	UP
Os07g0622200	Q75W16	Phospho-2-dehydro-3-deoxyheptonate aldolase	3.558	6.192	UP
Os02g0626100	P14717	Phenylalanine ammonia-lyase	3.596	4.920	UP
Os03g0192700	O64437	Inositol-3-phosphate synthase	5.191	5.754	UP
Os07g0681400	Q7XHW4	Probable calcium-binding protein	5.468	7.842	UP
Os06g0569500	Q0DBF4	Ent-sandaracopimaradiene 3-hydroxylase	6.003	7.766	UP
Os08g0536000	Q6Z1G7	Pyruvate dehydrogenase E1 component subunit	0.010	0.063	Down
Os11g0123400	A0A0N7KSC9	CTP:phosphoethanolamine cytidylyltransferase	0.010	0.010	Down
Os05g0595100	Q8LNZ3	UDP-glucose 4-epimerase	0.296	0.314	Down
Os02g0785800	Q6K8U8	Similar to Ribosomal protein L35A.	0.448	0.299	Down

## Data Availability

The original contributions presented in the study are included in the article/Supplementary Material, further inquiries can be directed to the corresponding author.

## Supplementary Materials

The following supporting information can be downloaded at: https://www.mdpi.com/article/10.3390/plants13172430/s1. Table S1. Genes ID of the 18S rRNA gene used in qRT-PCR in this experiment.
