# Supplementary material for: Physiological and Proteomic Analysis of Various Priming on Rice Seed under Chilling Stress
Source: Plants (Basel). 2024 Aug 30;13(17):2430. doi: 10.3390/plants13172430 (PMC11396840; doi:10.3390/plants13172430)
Supplement: Supplementary file 1 [file plants-13-02430-s001.zip › plants-3031698-supplementary.pdf]

Physiological and Proteomic Analysis of Various Priming on Rice Seed under Chilling Stress

Hua Zhang [1,2,3,†], Guo Hui [1,†], Guoqing Gao [1], Izhar Ali [2], Maoyan Tang [1], Lei Chen [1], Xiaoyuan Zhong [1], Ligeng Jiang [2], Tianfeng Liang [1,*] and Xiaoli Zhang [1,*]

Supplementary File

Table S1. Genes ID of the 18S rRNA gene used in qRT-PCR in this experiment.

gene ID	primer sequence
Os02t0626100-01F	GACCCTGTATTTTCTTCGTTCG
Os02t0626100-01R	AGTAGCAATACTTTCACCCCAA
Os03t0192700-01F	CAACAGGGAGGGAATCTCATG
Os03t0192700-01R	TAGATCTCCTCCCCGTTGTAG
Os04t0688200-01F	CATCACGTCAAAAGTCTTGTGT
Os04t0688200-01R	GTATTATCATGCACGCACGAG
Os08g0126300-00F	CCAAGACCCAGTAGAATCCTTT
Os08g0126300-00R	TCCCAACTAGCATTAACAGAGG
